# L'Amoebome colique: faut-il y penser plus souvent?

**DOI:** 10.11604/pamj.2013.14.136.2588

**Published:** 2013-04-08

**Authors:** Anis Benmansour, Aboubacar Traore, Rachid Bensaleh, Mohammed Amraoui, Rachid Chkoff

**Affiliations:** 1 Service d'urgences chirurgicales viscérales hôpital Avicennes Rabat, UPR de Chirurgie Générale. Faculté de médecine de Rabat, Maroc

**Keywords:** Amoebome, amibiase, colon, Amoeboma, amoebiasis, colon

## Abstract

L'amibiase est une parasitose encore très fréquente sous nos cieux. L'amoebome ‘pseudotumeur inflammatoire a *Entamoeba histolytica* - représente 1,5% des formes anatomo-cliniques de la maladie. Nous rapportons le cas d'un patient de 53 ans, sans antécédents, qui se présente aux urgences pour des douleurs de la fosse iliaque droite (FID). L'examen retrouvait un patient en bon état général, fébrile à 38,5 avec défense de la FID. Sur le plan biologique, on retrouvait une hyperleucocytose avec élévation de la Protéine C Réactive (CRP). Une échographie montrait une collection impure péri-appendiculaire. Le diagnostic d'abcès appendiculaire a été pose et l'indication opératoire retenue. L'intervention a débutée par une incision de Mac Burney qui a permis de retrouver une masse caecale. Une conversion en laparotomie médiane a été réalisée suivie d'une hémicolectomie droite avec anastomose iléo-colique. Les suites opératoires ont été simples. L’étude anatomo-pathologique de la pièce opératoire a révélée un amoebome caecal. A travers ce cas, nous tenons à reconsidérer les pièges diagnostiques et la conduite à tenir devant cette pathologie pseudotumorale colique; plus fréquente qu'on ne le pense.

## Introduction

L'amibiase est une parasitose encore très fréquente sous nos cieux. L'amoebome - pseudotumeur inflammatoire à *Entamoeba histolytica* - représente 1,5% des formes anatomo-cliniques de la maladie [1]. Ceci en fait une pathologie relativement fréquente quoique peu reconnue en particulier dans les zones d'endémie.

## Patient et observation

Nous rapportons le cas d'un patient de 53 ans, sans antécédents, qui se présente aux urgences pour des douleurs de la fosse iliaque droite (FID) isolées évoluant dans un contexte fébrile. L'examen à l'admission retrouvait un patient en bon état général, fébrile à 38,5 degrés avec défense au niveau de la FID. Le reste de l'abdomen était souple sans douleur provoquée ni masse palpable. Sur le plan biologique, on retrouvait une hyperleucocytose à 16000 éléments par ml à prédominance de neutrophiles avec élévation de la Protéine C Réactive (CRP).

Une échographie montrait une collection impure péri-appendiculaire. Le diagnostic d'abcès appendiculaire a été pose et l'indication opératoire retenue.

**Figure 1:**
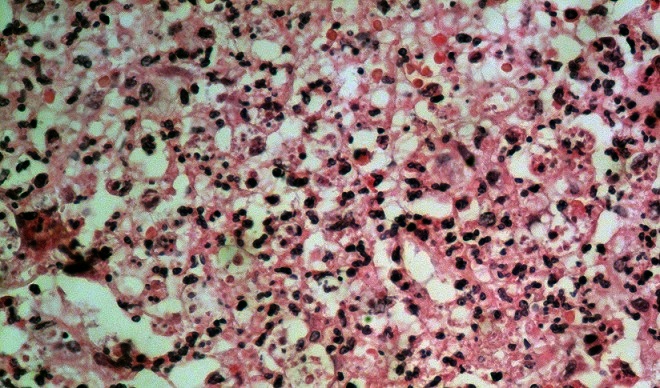
Image microscopique montrant les kystes d’*Entamoeba histolytica*

L'intervention a débutée par une incision de Mac Burney qui a permis de retrouver une masse caecale. Une conversion en laparotomie médiane a été réalisée suivie d'une hémicolectomie droite avec anastomose iléo-colique termino-laterale. Les suites opératoires ont été simples, avec un retour au domicile au sixième jour post-opératoire. L’étude anatomo-pathologique de la pièce opératoire a révélée à la macroscopie un épaississement pariétal prenant la base caecale, l'appendice et l'iléon. A la microscopie on retrouvait une muqueuse intestinale ulcérée par endroit et remplacée par un enduit fibrino-leucocytaire. Le chorion abritait un infiltrat inflammatoire dense arrivant jusqu′à la séreuse dans lequel on retrouvait des formations parasitaires correspondant à des amibes (Figure 1).

Le diagnostic d'amoebome caecal a donc été retenu. Un complément de traitement à base de métronidazole à la dose de 1,5g par jour reparti en trois prises pendant une durée de 10 jours a été instauré. Aucun contrôle ultérieur n'a été réalisé.

## Discussion

L'amibiase est une pathologie qui reste très fréquente quoique peu reconnue. Une étude épidémiologique nationale à grande échelle a révélé que deux personnes sur trois étaient infestées de parasites en milieu rural dont 50% par des amibes contre une sur deux en milieu urbain dont 33% d'amibes [2].si l'on considère que 1,5% des amibiases maladies se développeront sous forme d'amoebomes [1] cela rend compte de la fréquence de cette forme anatomo-clinique.

Sur le plan sémiologique, les diarrhées et les rectorragies viennent en premier lieu. Difficile alors de ne pas penser à un cancer colique lorsqu’ à côté de ses signes on retrouve à l'examen clinique une masse palpable sur le cadre colique. Lorsque cette masse s'associe à un abcès amibien hépatique, il est plus facile d’évoquer le diagnostic surtout lorsque l'on souhaite rester uniciste. Cependant devant ce tableau clinique et radiologique on peut également évoquer une tumeur colique métastasée au foie. Cela a était d'ailleurs rapporté dans la littérature [3].

Lorsque l'abcès amibien hépatique vient au premier plan, la constatation d'une masse sur le cadre colique est fortement évocatrice d'un amoebome. Cependant l’éclectisme doit être de règle. Ceci est démontré dans la série de MISRA et al [6] comprenant 17 masses iléo-caecales associées à des abcès amibiens hépatiques - il s'agit par ailleurs de la plus grande série d'amoebome connue à ce jour- Misra retrouve 14 amoebomes, 2 cas de tuberculose iléo-caecale et un cas d'adénocarcinome colique.

La leçon à retenir de cette série reste probablement qu'il ne faut éliminer un cancer colique qu'après preuve histologique avant et après traitement de l'amibiase. En effet nous insistons sur le contrôle endoscopique et histologique après traitement car le cancer colique peut être lui-même surinfecté par des amibes. Allah-kouadio rapporte le cas d'une femme de 50 ans qui consultait pour des rectorragies et chez qui la colonoscopie avait objectivée une nappe villeuse du sigmoïde. Les biopsies avaient révélées la présence d'amibes et la patiente a bien évoluée sous traitement médical. Le contrôle endoscopique à un mois retrouvait cette fois-ci une lésion ulcéro-bourgeonnante en rapport avec un adénocarcinome colique [4]. Ce cas illustre bien tout l'intérêt d'un contrôle endoscopique après traitement médical.

Dans notre observation, l'amoebome a mimé une urgence chirurgicale, ce qui est rarement rapporté dans la littérature. Dans ce contexte, Rebai [5] rapporte le cas d'un amoebome perforé opéré en urgence pris aux examens morphologiques pour une tumeur sigmoïdienne perforée. Le diagnostic n'ayant été redressé qu'après étude anatomo-pathologique de la pièce de sigmoïdectomie.

Sur le plan paraclinique, l'examen parasitologique des selles est souvent positif. En cas de négativité, la sérologie est positive dans 70 à 90% des cas. Les examens morphologiques, en particulier le scanner abdominal va retrouver un épaississement plus ou moins circonférentiel de la paroi colique. Il peut également montrer les abcès amibiens hépatiques éventuellement associés. L'aspect endoscopique peut être très évocateur d'un authentique carcinome en montrant une formation polypoïde pseudotumorale [3] ou une nappe villeuse [4].

L’étude histologique des biopsies faites au cours de la coloscopie ou des pièces opératoires va confirmer le diagnostic d'amoebome en montrant des lésions de fibroses obturant la lumière intestinale et fusant dans les mésos. A la microscopie cette fibrose est faite de granulations inflammatoires a prédominance lympho-plasmocytaire et éosinophile avec des abcès ou sont retrouvés des amibes.

Au total, ni la clinique ni la paraclinique ne sont spécifiques d'un amoebome. Surtout que la présence de parasites n’élimine pas une néoplasie sous-jacente associée. Le diagnostic de certitude n'est apporté que par les biopsies, répétées après traitement médical de l'amibiase pour éliminer de manière formelle une pathologie associée dont le traitement et le pronostic diffèrent.

## Conclusion

L'amoebome -forme pseudo-tumorale- de l'amibiase est un diagnostic différentiel du cancer colique qu'il faut évoquer devant toute masse colique surtout que nous nous trouvons en zone d'endémie. Les examens morphologiques et l'aspect endoscopique n’étant pas spécifiques de l'une ou de l'autre, le diagnostic d'amoebome ne sera retenu qu'après étude anatomo-pathologique avant et après traitement médical de l'amibiase. En effet cette démarche diagnostique bien conduite, éviterai une chirurgie en cas d'amoebome accessible au traitement médical mais éviterai également de prendre pour amoebome une authentique tumeur colique surinfectée d'amibes.
